# Blood RNA biomarkers and C-reactive protein for triage of adult patients with tuberculosis lymphadenitis and pericarditis in South Africa: a single-centre, prospective, observational, diagnostic accuracy study

**DOI:** 10.1016/j.lanmic.2025.101153

**Published:** 2025-07-14

**Authors:** Tiffeney Mann, Stephanie Minnies, Rishi K Gupta, Byron W P Reeve, Georgina Nyawo, Zaida Palmer, Charissa Naidoo, Anton Doubell, Alfonso Pecoraro, Thadathilankal-Jess John, Pawel Schubert, Claire J Calderwood, Aneesh Chandran, Grant Theron, Mahdad Noursadeghi

**Affiliations:** Division of Infection and Immunity, University College London, London, UK; DSI-NRF Centre of Excellence for Biomedical Tuberculosis Research, South African Medical Research Council Centre for Tuberculosis Research, Division of Molecular Biology and Human Genetics, Faculty of Medicine and Health Sciences, Stellenbosch University, Cape Town, South Africa; UCL Respiratory, Division of Medicine, University College London, London, UK; DSI-NRF Centre of Excellence for Biomedical Tuberculosis Research, South African Medical Research Council Centre for Tuberculosis Research, Division of Molecular Biology and Human Genetics, Faculty of Medicine and Health Sciences, Stellenbosch University, Cape Town, South Africa; Department of Medicine, Division of Cardiology, Stellenbosch University and Tygerberg Academic Hospital, Cape Town, South Africa; National Health Laboratory Service, Tygerberg Hospital, Cape Town, South Africa; Division of Anatomical Pathology, Faculty of Medicine and Health Sciences, Stellenbosch University, Cape Town, South Africa; Department of Clinical Research, Faculty of Infectious and Tropical Diseases, London School of Hygiene & Tropical Medicine, London, UK; Division of Infection and Immunity, University College London, London, UK; Institute of Dentistry, Queen Mary University London, London, UK; DSI-NRF Centre of Excellence for Biomedical Tuberculosis Research, South African Medical Research Council Centre for Tuberculosis Research, Division of Molecular Biology and Human Genetics, Faculty of Medicine and Health Sciences, Stellenbosch University, Cape Town, South Africa; Division of Infection and Immunity, University College London, London, UK

## Abstract

**Background:**

Data on the diagnostic accuracy of blood RNA biomarker signatures for extrapulmonary tuberculosis are scarce. We aimed to address this question among people investigated for tuberculosis lymphadenitis and tuberculosis pericarditis.

**Methods:**

This prospective, observational, diagnostic accuracy study was done at a tertiary hospital in Cape Town, South Africa. We enrolled consecutive symptomatic adults (aged 18 years or older) with presumptive tuberculosis lymphadenitis (Jan 25, 2017, to Oct 9, 2019) or tuberculosis pericarditis (Nov 24, 2016, to Oct 28, 2019). We used microbiological testing of samples from the site of disease as the reference standard. We evaluated the diagnostic accuracy of seven previously reported blood RNA signatures by area under the receiver operating characteristic curve (AUROC) and sensitivity and specificity at prespecified thresholds using two SDs above the mean of a healthy reference control group, benchmarked against blood C-reactive protein and WHO target product profile for a tuberculosis triage test. Decision curve analysis was used to evaluate clinical utility of the best-performing blood RNA signature and C-reactive protein.

**Findings:**

The pooled cohort included 440 individuals, 374 of whom (275 with lymphadenitis and 99 with pericarditis) had at least one microbiological test from the site of disease, blood C-reactive protein, and RNA measurements available and were included in the analysis. 181 (48%) participants were female and 193 (52%) were male. The diagnostic accuracy of blood RNA signatures was similar across patients with lymphadenitis and pericarditis. In pooled analysis of both cohorts, all RNA signatures had similar discrimination, with AUROC point estimates ranging from 0·77 (95% CI 0·72–0·82) to 0·82 (0·77–0·86), and greater than that of C-reactive protein (0·61 [0·56–0·67]). The best-performing signature (Roe3) did not meet the WHO target product profile benchmark for a triage test. At the prespecified threshold, Roe3 had 78% (95% CI 72–83) sensitivity and 69% (62–75) specificity; C-reactive protein at a threshold of 10 mg/L had 83% (77–87) sensitivity and 35% (29–43) specificity. In this setting, decision curve analysis showed that Roe3 offered greater net benefit than other approaches for services aiming to reduce the number needed to investigate with confirmatory testing to fewer than four to identify each individual with tuberculosis.

**Interpretation:**

Our results suggest RNA biomarkers show better accuracy and clinical utility than C-reactive protein to trigger confirmatory tuberculosis testing in patients with tuberculosis lymphadenitis and tuberculosis pericarditis, but still fall short of the WHO target product profile for tuberculosis triage tests.

**Funding:**

South African Medical Research Council, European and Developing Countries Clinical Trials Partnership 2, National Institutes of Health/National Institute of Allergy and Infectious Diseases, Wellcome Trust, National Institute for Health and Care Research, and Royal College of Physicians.

## Introduction

Delays in the diagnosis of tuberculosis remain a substantial barrier to reducing the associated morbidity and mortality for individuals as well as effectiveness of worldwide tuberculosis control programmes at the population level.^1^ It is estimated that extrapulmonary disease comprises approximately 10–30% of new or relapsed tuberculosis cases.^1,2^ Although tuberculosis incidence has slowly declined over the past 20 years, extrapulmonary tuberculosis incidence has plateaued.^2–6^ Microbiological diagnosis of tuberculosis has mostly relied on sampling the site of disease. In pulmonary tuberculosis, non-invasive sputum expectoration can provide appropriate samples for microbiological diagnosis. Diagnosis of extrapulmonary tuberculosis remains more challenging because of the need for invasive procedures to sample the site of disease, necessitating access to hospitals with specialised infrastructure and expertise that are resource intensive. Additionally, confirmatory molecular tests for tuberculosis have lower sensitivity in extrapulmonary tuberculosis than pulmonary tuberculosis.^7,8^ These barriers have the greatest effect in low-income and middle-income countries (LMICs) where extrapulmonary tuberculosis incidence might be systematically underestimated, particularly among people living with HIV who have higher risk of extrapulmonary tuberculosis than the general population.^9,10^ To address this limitation in both extrapulmonary tuberculosis and sputum-scarce pulmonary tuberculosis, and to mitigate against the health economic barriers to systematic microbiological testing, WHO has recommended development of sensitive, cheap, point-of-care blood-based triage tests to enable more precise targeting of patients for confirmatory diagnostic testing.^11^

Blood RNA biomarkers and C-reactive protein measurements have emerged as leading candidates for blood-based triage tests for tuberculosis.^12,13^ C-reactive protein is a hepatocyte-derived acute phase protein and increased concentrations in the circulation are induced by IL-6 and other acute phase cytokine activity. C-reactive protein is most widely used as a non-specific biomarker of bacterial infections.^14^ The advent of a cheap, quantitative, point-of-care test for C-reactive protein has made it particularly attractive as a triage test for tuberculosis in LMIC settings, where it has been evaluated for screening for pulmonary tuberculosis in people living with HIV before starting antiretroviral therapy (ART)^15^ and among people presenting with tuberculosis symptoms.^16^ The diagnostic accuracy of C-reactive protein for extrapulmonary tuberculosis has, to our knowledge, not been tested.

Numerous candidate blood RNA biomarkers of tuberculosis have been reported, ranging from single-gene transcripts to multigene signatures. We have shown that the best performing blood RNA biomarkers of tuberculosis are co-correlated and reflect canonical host immune responses downstream of TNF and IFN signalling.^17^ Although previous diagnostic accuracy studies of blood RNA biomarkers for tuberculosis have included people with extrapulmonary tuberculosis, they were not powered to evaluate differences in biomarker accuracy between pulmonary and extrapulmonary disease. To our knowledge, no studies have evaluated diagnostic accuracy exclusively among people with signs and symptoms suggestive of extrapulmonary tuberculosis.

Extrapulmonary tuberculosis can affect any organ system and tuberculosis lymphadenitis is among the most common extrathoracic sites of disease.^2–4^ Tuberculosis pericarditis is less common but provides a prototypic example of the association of extrapulmonary tuberculosis with HIV co-infection.^18,19^ We therefore did a head-to-head comparison of the best-performing candidate RNA biomarkers from our previous studies^12,17^ among people being evaluated for tuberculosis lymphadenitis or pericarditis at a referral centre in a hyperendemic setting for tuberculosis and HIV infection. We aimed to benchmark RNA biomarker performance against C-reactive protein and the WHO target product profile for a blood-based triage test aiming to achieve sensitivity of 90% or more and specificity of 70% or more.^11^ We also aimed to evaluate comparative clinical utility by decision curve analysis.

## Methods

### Study design and participants

This prospective, observational, diagnostic accuracy study was done at Tygerberg Academic Hospital, a tertiary hospital in Cape Town, South Africa. Consecutive adults aged 18 years and older who were referred for routine investigation by invasive sampling for symptomatic tuberculosis lymphadenitis from Jan 25, 2017, to Oct 9, 2019, or for tuberculosis pericarditis from Nov 24, 2016, to Oct 28, 2019 were recruited. For the lymphadenitis cohort, patients who had received tuberculosis treatment within 2 months before presentation were excluded. For the pericarditis cohort, we included people up to 2 weeks after tuberculosis treatment initiation. Subsets of the patients included in the present study contributed to a diagnostic accuracy study of Xpert MTB/RIF Ultra (Ultra) in tuberculosis lymphadenitis,^20^ and evaluation of microbiota in tuberculosis lymphadenitis^21^ or tuberculosis pericarditis.^22^ All participants provided written informed consent.

The study was approved by the Stellenbosch University Faculty of Health Sciences Research Ethics Committee (N16/04/050) and the Western Cape Department of Health, South Africa (WC_2016RP15_762). The study is reported in line with standards for reporting diagnostic accuracy studies guidance.^23^

### Procedures

Demographic characteristics, laboratory-confirmed HIV status and self-reported tuberculosis treatment (within 12 weeks of enrolment) were recorded for all participants. Sex was self-reported as male or female. In both the lymphadenitis cohort and the pericarditis cohort, diagnostic testing on samples from the site of disease included Xpert MTB/RIF (Xpert) or Ultra, with or without liquid culture for *Mycobacterium tuberculosis*, and cytology performed on lymph node aspirates according to predefined algorithms (appendix 1 p 3). For both cohorts, the results from sputum smear microscopy, *M tuberculosis* culture, and molecular testing on other non-site-of disease fluids conducted as part of routine care were also recorded where available. Except for site-of-disease Ultra testing, all other investigations were performed as part of routine care.

Serum and blood RNA samples (collected in Tempus tubes; Ambion Life Technologies, Austin, TX, USA) were taken at recruitment and stored at −80°C. Seven concise blood RNA signatures selected from the best-performing biomarkers of tuberculosis in our previous comparative studies^12,17^ were measured using the Nanostring platform as described previously.^24^ Each signature was named using the first author’s surname from the original publication where it was derived followed by the number of component genes, except for BATF2 (a single transcript) and RISK6 (named by the original investigators). These included the following signatures: BATF2,^25^ Gliddon3,^26^ RISK6,^27^ Roe3,^28^ Suliman4,^29^ Sweeney3,^30^ and Darboe11 (derived from Zak16).^31^ Blood RNA signature Z scores were calculated by subtracting the mean and dividing by the SD of blood RNA samples (n=48) from a multiethnic healthy control population of individuals with latent tuberculosis,^24,32^ measured using the same Nanostring code-set batches. This group was used as a control population because we were able to exclude subclinical tuberculosis by chest radiography as part of their routine assessment, and because their previous immune sensitisation is more representative of the general adult population in the high-burden setting of our study, which has very high prevalence rates of past *M tuberculosis* infection.

All samples were processed across two code-set batches. Principal component analysis of reference RNA samples (using the prcomp() function in R) and distributions of the processed gene-level data across the two code-set batches suggested a small batch effect (appendix 1 p 4). We therefore performed batch correction with the ComBat function from the sva package in our primary analyses, as previously described^24^ (appendix 1 pp 5–6). Serum C-reactive protein was measured on biobanked serum using the Cobas high-sensitive immunoturbidimetric assay (Roche Diagnostics Limited, Burgess Hill, UK).

We used a composite microbiological reference standard for tuberculosis in both cohorts comprising positive *M tuberculosis* culture or molecular test (Xpert or Ultra, excluding trace results) on samples from the site of disease or sputum. In sensitivity analyses, we examined a range of alternative reference standards where, in addition to the microbiological tests details described, we included Ultra trace results as positive; included patients commenced on tuberculosis treatment within 12 weeks after study enrolment as tuberculosis-positive; and included cytology or histology consistent with tuberculosis, as defined by evidence of necrotising granulomas.

### Statistical analysis

The sample size of each cohort was primarily determined by the number of participants in the parent studies for which blood RNA, C-reactive protein, and reference standard results were available. We calculated statistical precision in Power Analysis and Sample Size Software 2024 (National Council for Social Studies), using published models for estimates of sample size calculations in diagnostic tests.^33,34^ Our sample sizes provided more than 80% power to test whether each biomarker could achieve the WHO target product profile thresholds^11^ of 90% sensitivity with a 5% CI and 70% specificity with 10% CI (appendix 1 p 7).

All analyses were conducted using R (version 4.0.2). From the parent cohorts, we excluded participants without RNA, C-reactive protein, or microbiology test results required for the reference standard, representing a complete case analysis approach. In our primary analysis, we quantified area under the receiver operating characteristic curve (AUROC) and 95% CIs^35^ for each biomarker and for C-reactive protein to discriminate between patients with and without tuberculosis across both clinical syndromes, since we observed similar performance in each cohort. We compared AUROCs for each RNA signature with C-reactive protein using paired DeLong tests, with adjustment for multiple testing using a Benjamini–Hochberg correction. Sensitivity, specificity, and predictive values for each biomarker were calculated at different test thresholds: two SDs above the mean of the healthy latent tuberculosis control population (Z2) for the RNA signatures, 10 mg/L for C-reactive protein, and at the Youden Index of each receiver operating characteristic curve. To benchmark test performance against the WHO target product profile,^11^ we also calculated the specificity and predictive value of each biomarker at thresholds that achieved 90% sensitivity. 95% CIs were calculated using the DeLong method for AUROCs and Wilson method for proportions, as previously described.^12^

In secondary analyses, we evaluated biomarker performance stratified by disease site. We also performed subgroup analyses by HIV status, and by HIV treatment status for participants with HIV. The clinical utility of candidate tuberculosis screening strategies was assessed by decision curve analysis using the rmda package as previously described^13,24^ to evaluate net benefit for the best-performing blood RNA biomarker (at a threshold of Z2) to guide invasive confirmatory microbiological testing, compared with confirmatory testing for all, confirmatory testing for none, and confirmatory testing guided by C-reactive protein (at thresholds of ≥5 mg/L and ≥10 mg/L). In addition to the alternative reference standard definitions, we also performed a sensitivity analysis without batch correction.

### Role of the funding source

The funders of the study had no role in study design, data collection, data analysis, data interpretation, or writing of the report.

## Results

**Figure 1:**
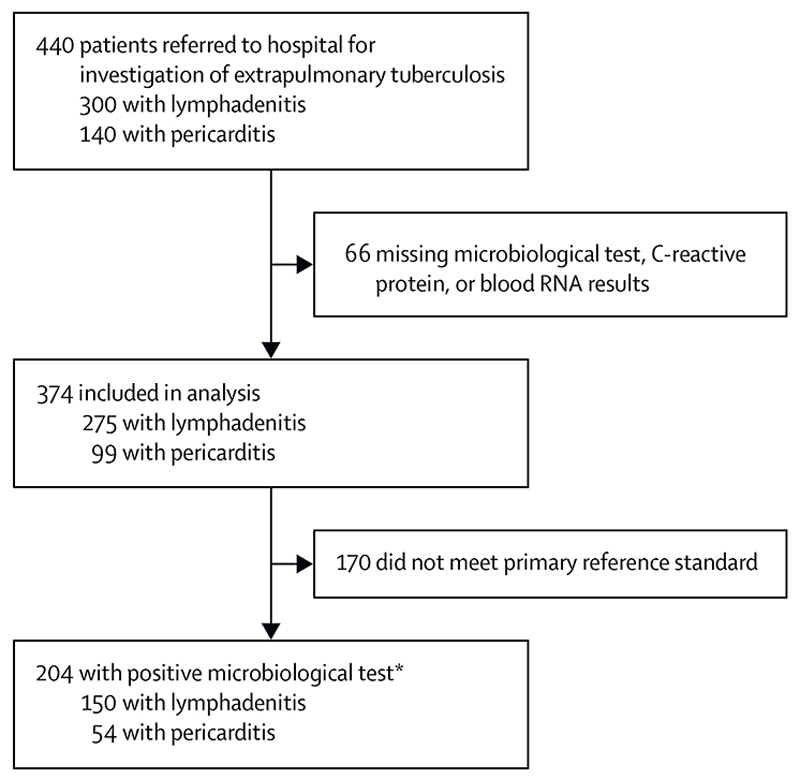
Study flow diagram 170 patients included in the analysis did not meet the primary reference standard; however, 25 had trace Xpert MTB/RIF Ultra results, 20 had cytology or histology consistent with tuberculosis, and 43 were started on tuberculosis treatment within 12 weeks of enrolment and were included in the sensitivity analyses. *Positive *Mycobacterium tuberculosis* culture or molecular test (Xpert MTB/RIF or Xpert MTB/RIF Ultra, excluding trace results) on samples from the site of disease or sputum.

The pooled cohort comprised 440 individuals, of whom 66 people were excluded because they had any one of missing microbiological (n=2), C-reactive protein (n=6), or RNA (n=58) results (appendix 1 p 15). The excluded participants were generally similar to those included in the analysis (appendix 1 p 15). Among the 374 participants included in the analysis, 275 had lymphadenitis and 99 had pericarditis (table 1, figure 1). Median age was 38 years (IQR 31–48), 181 (48%) of 374 were female, 193 (52%) were male, and 200 (53%) were people living with HIV (95 [48%] of 198 with known ART status were receiving ART at enrolment).

204 (55%) of 374 participants met the primary reference standard for tuberculosis of a positive culture or molecular test for *M tuberculosis* from the site of disease or sputum. Of these, 20 (10%) participants had positive *M tuberculosis* sputum culture or sputum molecular tests. The prevalence of microbiologically confirmed tuberculosis was 150 (55%) of 275 participants with lymphadenitis, and 54 (55%) of 99 participants with pericarditis. In the lymphadenitis cohort, the majority of participants with known site of disease (199 [73%] of 273) had cervical lymphadenitis (appendix 1 p 15), of whom 122 (61%) met the primary reference standard. In the pericarditis cohort, only 12 (12%) of 99 participants had started tuberculosis treatment at the time of recruitment, precluding evaluation of the effect of previous treatment on biomarker diagnostic accuracy.

**Figure 2:**
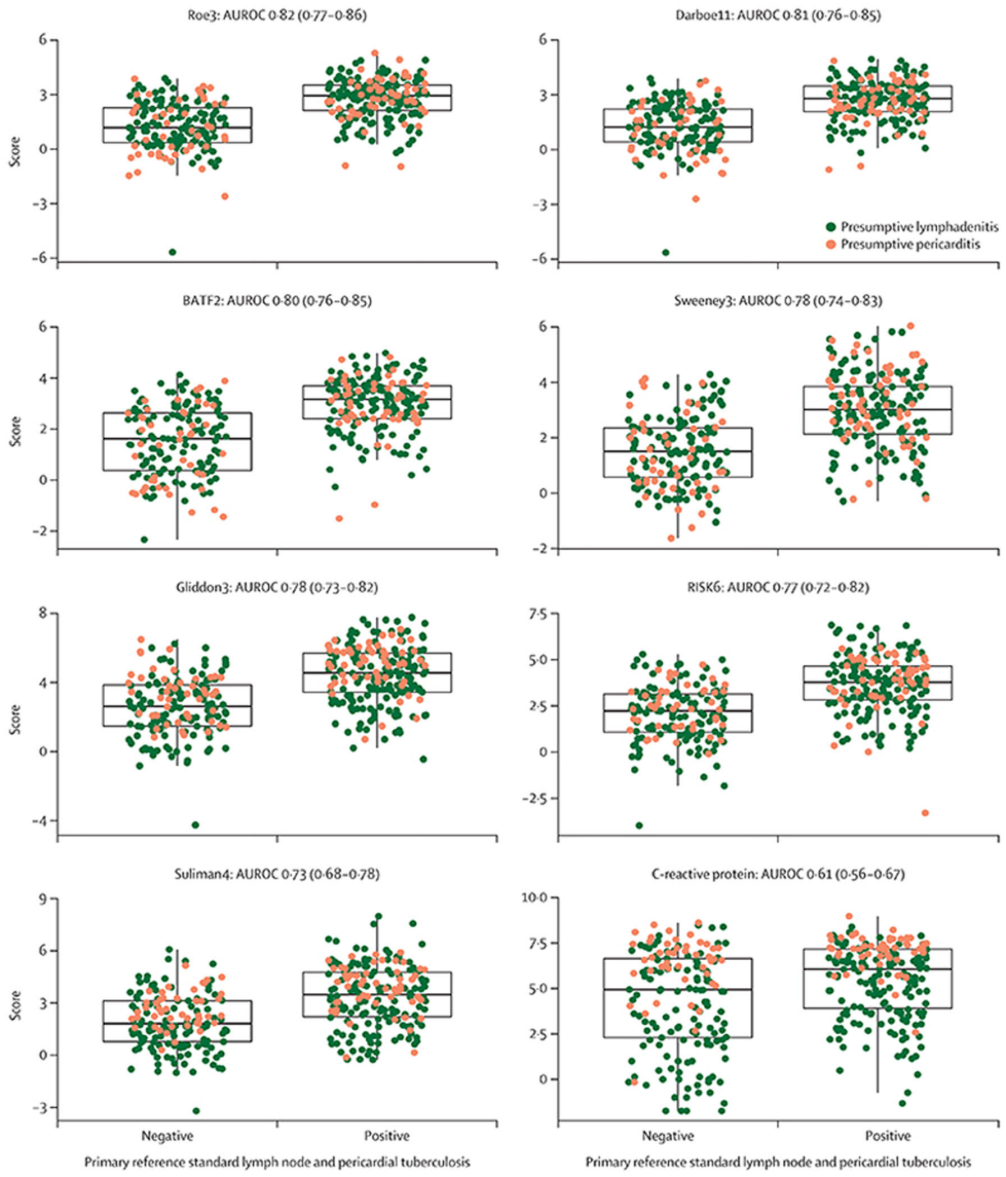
Blood RNA biomarker discrimination of primary reference standard for lymph node and pericardial tuberculosis Scores shown as Z scores for RNA signatures, and log-2 transformed C-reactive protein (mg/L), stratified by disease cohort. Discrimination of the pooled dataset presented as AUROC, with 95% CIs. AUROC=area under the receiver operating characteristic curve.

In the primary analysis, all blood RNA biomarkers showed similar diagnostic accuracy to identify participants with microbiologically confirmed tuberculosis (figure 2, table 2). All the blood RNA biomarkers showed significantly better AUROC than C-reactive protein, with adjusted p values of less than 0·05. The Roe3 signature showed the highest AUROC point estimate of 0·82 (95% CI 0·77–0·86; table 2). By comparison, C-reactive protein had an AUROC of 0·61 (0·56–0·67; table 2). At predefined Z2 test thresholds for the blood RNA signatures, Roe3 showed 78% (95% CI 72–83) sensitivity and 69% (62–75) specificity (table 2). A predefined test threshold of 10 mg/L for C-reactive protein, which we have assessed previously as a triage test for pulmonary tuberculosis,^13^ had a sensitivity of 83% (77–87) and specificity of 35% (29–43; table 2). When fixing the sensitivity of each test to 90% (in line with the WHO target product profile), Roe3 had 52% (45–60) specificity and C-reactive protein had 27% (21–34) specificity, both below the WHO specificity target of 70% (appendix 1 p 16).

In secondary analyses, we evaluated the performance of C-reactive protein and blood RNA biomarkers stratified by disease cohort. We found their performance to be similar in lymphadenitis and pericarditis (appendix 1 pp 8–9, 17–18). Of note, among participants with tuberculosis, the C-reactive protein concentrations in the pericarditis cohort were significantly higher than those in the lymphadenitis cohort (appendix 1 p 10). This difference was less evident among the blood RNA signatures (appendix 1 p 10). Biomarker diagnostic accuracy using alternate reference standards was similar to that of the primary analysis (appendix 1 pp 11–14). The point estimates for biomarker diagnostic accuracy among people living with HIV were lower than those without HIV infection but these differences were not statistically significant (appendix 1 p 19).

**Figure 3:**
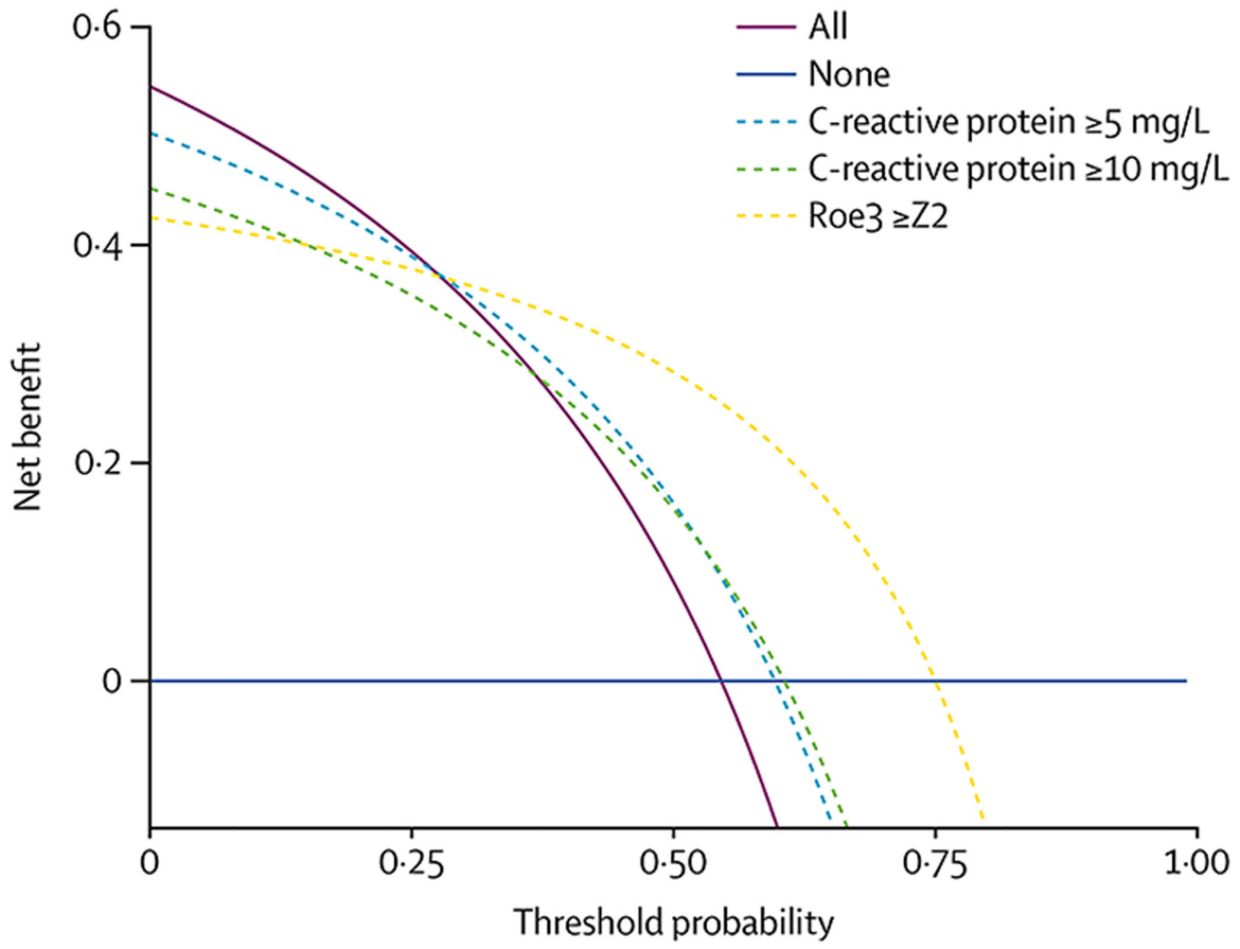
Decision curve analysis for alternative triage strategies to trigger confirmatory investigations for tuberculosis Net benefit (true positive rate − false positive rate weighted by cost–benefit ratio) for investigate all and investigate none approaches across the range of threshold probabilities that a service might use to trigger confirmatory investigations for tuberculosis compared with that of decisions to investigate triggered by each triage strategy. Z2=two SDs above the mean of the healthy latent tuberculosis control population.

In addition, we used the pooled dataset to evaluate clinical utility of Roe3 and that of C-reactive protein using decision curve analysis, benchmarked against the alternative approaches of undertaking confirmatory diagnostic investigations in all or none.^36^ This analysis quantifies the net benefit of a given approach, representing the trade-off between the true positive rate and a weighted false positive rate across a range of test threshold probabilities that trigger an intervention. The test threshold probabilities (reflecting the perceived cost–benefit ratio that triggers an intervention) are used for weighting the false positive rate. The intervention triggered by a triage test would be to proceed to confirmatory diagnostic investigations. Therefore, the triage test threshold probability can be considered as the number willing to investigate to identify each tuberculosis case. In our evaluation of clinical utility, the Roe3 blood RNA biomarker (>Z2) showed greater net benefit than confirmatory testing for all at a test threshold probability greater than 0·28. By comparison, C-reactive protein (>10 mg/L) provided greater net benefit than confirmatory testing for all at a test threshold probability greater than 0·37 (figure 3). These data suggest that using blood RNA biomarkers to direct use of confirmatory testing provides the best approach for comparable services in HIV and tuberculosis hyperendemic settings where the number willing to test by confirmatory diagnostics is fewer than four for each case of extrapulmonary tuberculosis. In settings willing to test more than four people with a confirmatory test for each true case of extrapulmonary tuberculosis, a test-all approach achieves greater net benefit than a triage approach using any of the biomarkers tested in the present study.

## Discussion

To the best of our knowledge, this is the first diagnostic accuracy study of blood RNA biomarkers focusing on extrapulmonary tuberculosis. We undertook our study in a hyperendemic setting for both tuberculosis and HIV. In a pooled dataset of peopleunderinvestigation for tuberculosis lymphadenitis or pericarditis, all the blood RNA biomarkers tested had similar performance, with similar AUROCs as previously reported for pulmonary tuberculosis.^12,37^ For most biomarkers, blood RNA signatures showed higher point estimates for accuracy in people without HIV compared to in people living with HIV, but with overlapping 95% CIs. Additionally, we undertook head-to-head comparison of blood RNA biomarkers with serum C-reactive protein concentrations. In contrast with previous reports, in which C-reactive protein measurements achieve similar diagnostic accuracy to blood RNA biomarkers in pulmonary tuberculosis,^13,24^ we found C-reactive protein was significantly inferior to blood RNA biomarkers for the extrapulmonary tuberculosis syndromes tested here, both among people living with HIV and people without HIV.

Importantly, sputum tests identified less than 10% of the participants with tuberculosis in our pooled analysis of tuberculosis lymphadenitis and pericarditis, often because sputum acquisition was not attempted. Given that people with extrapulmonary tuberculosis might have concomitant pulmonary tuberculosis, this finding highlights the importance of strengthening sputum collection in addition to sampling from the site of disease in extrapulmonary tuberculosis. Therefore, we envisioned the primary application of biomarkers of extrapulmonary tuberculosis as triage tests to prioritise use of invasive sampling for confirmatory testing. The application of such tests in a primary care setting might prioritise patients for referral to secondary care, reducing costs and attrition across the cascade of care. In this context, none of the biomarkers tested met the combined sensitivity of more than 90% and specificity of more than 70% recommended by WHO.^11^ Nonetheless, we assessed their potential clinical utility by decision curve analysis. The best-performing blood RNA biomarker showed greater net benefit than either C-reactive protein or unrestricted approaches to trigger invasive confirmatory testing for services aiming to reduce the number needed to investigate to fewer than four to identify each individual with tuberculosis. In settings with such high prevalence of tuberculosis and, therefore, high pre-test probability of tuberculosis, unrestricted approaches to confirmatory testing might be preferable. Settings with lower pre-test probability of tuberculosis might be expected to derive greater benefit from triage tests.

In our secondary analyses, we showed that the performance of the biomarkers was equivalent in tuberculosis lymphadenitis and pericarditis. In addition, the use of alternative reference standards did not substantially alter our results. We focused on selected blood RNA biomarkers, previously identified by systematic review, which showed the best performance for diagnosis of preclinical or incipient and symptomatic tuberculosis and were sufficiently concise to undertake multiplex measurements using a validated Nanostring platform.^17,24,37^ We have previously shown in a diagnostic accuracy study for pulmonary tuberculosis among people living with HIV that these RNA biomarkers show greater correlation with each other than with C-reactive protein,^24^ suggesting that tuberculosis-associated blood transcriptional changes are regulated differently to that of C-reactive protein concentrations. This interpretation is consistent with the findings in the present study showing that C-reactive protein does not identify individuals with extrapulmonary tuberculosis as well as blood RNA biomarkers. It is interesting that C-reactive protein concentrations were substantially higher in participants with tuberculosis pericarditis than tuberculosis lymphadenitis. This difference was absent or less significant among the blood RNA signatures, suggesting a hierarchy for recruitment of immunological pathways with increasing inflammatory manifestations of disease. We propose that TNF and IFN signalling, which we previously identified as drivers of the blood RNA biomarkers of tuberculosis,^17^ are more conserved across the spectrum of tuberculosis disease sites than the immunological pathways that regulate C-reactive protein, which are more strongly linked to IL-6 signalling.^38^ If so, then despite recent interest in adopting point-of-care C-reactive protein testing as a triage test for tuberculosis,^13,16,39^ we might expect that blood RNA bio-markers provide a more consistent performance for the range of disease manifestations beyond pulmonary tuberculosis. IL-6 signalling has also been associated with a risk of tuberculosis^40^ and its immunopathogenesis,^32^ consistent with the finding that C-reactive protein concentrations are higher in individuals with tuberculosis pericarditis, reflecting a more severe variant of disease than tuberculosis lymphadenitis.

The strengths of our study include robust classification of tuberculosis based on highly specific microbiological tests in the primary analysis, complemented by sensitivity analyses using alternative reference standards; head-to-head comparison of multiple blood RNA signatures with C-reactive protein; inclusion of two distinct extrapulmonary tuberculosis syndromes; and adequate sample sizes to evaluate the performance of biomarkers with a good level of precision.

Our study also has important limitations. The single-site design might limit the generalisability of our findings, particularly for settings with lower prevalence of tuberculosis and HIV. Future studies of extrapulmonary tuberculosis in different settings will be required to address this limitation. Settings with lower pre-test probability of tuberculosis, for example, earlier in the cascade of care or settings with lower prevalence, might have much greater differential clinical utility between biomarker triage and unrestricted approaches to confirmatory testing for extrapulmonary tuberculosis. Additionally, we cannot confidently generalise our findings across the full spectrum of extrapulmonary tuberculosis diseases. The similarities in RNA biomarker performance between tuberculosis lymphadenitis and tuberculosis pericarditis is a valuable starting point, but future studies will require testing of a wider range of extrapulmonary tuberculosis syndromes.

Notwithstanding these limitations, our study substantially extends our insights into the real-world evaluation of the leading candidates for tuberculosis diagnostic triage tests by providing the largest available dataset for extrapulmonary tuberculosis. Next-generation blood RNA biomarkers show some clinical utility. They still fall short of the desired accuracy targets and will require comprehensive health economic evaluation to establish the costs at which triage tests can confer a substantial advantage. Importantly, blood C-reactive protein measurements show significantly inferior accuracy to blood RNA biomarkers in extrapulmonary tuberculosis, and to the previously reported accuracy in pulmonary tuberculosis. We conclude that C-reactive protein-based triage is unlikely to be useful in this context. Multivariable models incorporating clinical, epidemiological, and laboratory measurements are urgently needed to overcome the limitations of individual biomarker approaches, alongside further innovation in discovery of novel diagnostic biomarkers.

## Supplementary Material

Supplementary appendix 1

Supplementary appendix 2

## Figures and Tables

**Table 1:** Summary characteristics of lymphadenitis and pericarditis cohorts

	Overall (n=374)	Lymphadenitis (n=275)	Pericarditis (n=99)
Age, years	38 (31-48)	38 (31-46)	37 (30-49)
Sex			
Female	181 (48%)	138 (50%)	43 (43%)
Male	193 (52%)	137 (50%)	56 (57%)
Previous tuberculosis			
Yes	91 (24%)	70 (25%)	21 (21%)
No	281 (75%)	203 (74%)	78 (79%)
Missing	2 (1%)	2 (1%)	0
HIV status			
Negative	172 (46%)	131 (48%)	41 (41%)
Positive	200 (53%)	142 (52%)	58 (59%)
Missing	2 (1%)	2 (1%)	0
On antiretroviral therapy*			
Yes	95 (48%)	75 (53%)	20 (34%)
No	103 (52%)	65 (46%)	38 (66%)
Missing	2 (1%)	2 (1%)	0
CD4, cells/μL*	158 (49-288)	158 (46-272)	176 (52-332)
Missing	1 (1%)	0	1 (2%)
CD4 <200 cells/μL*	118 (59%)	84 (59%)	34 (60%)
Missing	1 (1%)	0	1 (2%)
Number of molecular tests performed on samples from the site of disease			
0	2 (1%)	2 (1%)	0
1	156 (42%)	156 (57%)	0
2	124 (33%)	117 (43%)	7 (7%)
3	64 (17%)	0	64 (65%)
4	28 (7%)	0	28 (28%)
Fluid Xpert MTB/RIF or Xpert MTB/RIF Ultra			
Negative	154 (41%)	117 (43%)	37 (37%
Positive	218 (58%)	156 (57%)	62 (63%)
Not done	2 (1%)	2 (1%)	0
Fluid culture			
Negative	174 (47%)	121 (44%)	53 (54%)
Positive	84 (22%)	42 (15%)	42 (42%)
Not done	116 (31%)	112 (41%)	4 (4%)
Biopsy culture			
Negative	8 (2%)	ND	8 (8%)
Positive	13 (3%)	ND	13 (13%)
Not done	353 (94%)	275 (100%)	78 (79%)
Cytology			
Consistent with tuberculosis	126 (34%)	126 (46%)	0
Not consistent with tuberculosis	220 (59%)	133 (48%)	87 (88%)
Not done	28 (7%)	16 (6%)	12 (12%)
Histology			
Consistent with tuberculosis	9 (2%)	ND	9 (9%)
Not consistent with tuberculosis	14 (4%)	ND	14 (14%)
Not done	351 (94%)	275 (100%)	76 (77%)
Sputum culture or molecular			
Negative	39 (10%)	22 (8%)	17 (17%)
Positive	20 (5%)	13 (5%)	7 (7%)
Not done	315 (84%)	240 (87%)	75 (76%)
Primary reference standard			
Negative	170 (45%)	125 (45%)	45 (45%)
Positive	204 (55%)	150 (55%)	54 (55%)
Secondary reference standard including trace Xpert MTB/RIF Ultra			
Negative	145 (39%)	110 (40%)	35 (35%)
Positive	229 (61%)	165 (60%)	64 (65%)
Secondary reference standard including cytology or histology consistent with tuberculosis			
Negative	150 (40%)	106 (39%)	44 (44%)
Positive	224 (60%)	169 (61%)	55 (56%)
Secondary reference standard including tuberculosis treatment initiation within 3 months			
Negative	127 (34%)	107 (39%)	20 (20%)
Positive	247 (66%)	168 (61%)	79 (80%)

Data are median (IQR) or n (%). *Limited to HIV-positive participants. ND=not done.

**Table 2:** Diagnostic accuracy of blood RNA biomarkers and C-reactive protein for tuberculosis lymphadenitis and pericarditis

	AUROC	Sensitivity	Specificity	PPV	NPV	Triage positive	NNT each positive case	NNT each negative case
Roe3	0·82 (0·77–0·86)	0·78 (0·72–0·83)	0·69 (0·62–0·75)	0·75 (0·69–0·80)	0·72 (0·65–0·79)	0·57 (0·52–0·62)	1·3 (1·2–1·5)	3·6 (2·8–4·7)
Darboe11	0·81 (0·76–0·85)	0·77 (0·71–0·83)	0·66 (0·59–0·73)	0·73 (0·67–0·79)	0·71 (0·64–0·78)	0·57 (0·52–0·62)	1·4 (1·3–1·5)	3·5 (2·7–4·5)
BATF2	0·80 (0·76–0·85)	0·83 (0·77–0·87)	0·59 (0·51–0·66)	0·71 (0·65–0·76)	0·74 (0·66–0·81)	0·64 (0·59–0·69)	1·4 (1·3–1·5)	3·9 (2·9–5·2)
Sweeney3	0·78 (0·74–0·83)	0·76 (0·7–0·82)	0·65 (0·57–0·71)	0·72 (0·66–0·78)	0·70 (0·62–0·76)	0·58 (0·53–0·63)	1·4 (1·3–1·5)	3·3 (2·6–4·2)
Gliddon3	0·78 (0·73–0·82)	0·92 (0·87–0·95)	0·37 (0·30–0·45)	0·64 (0·58–0·69)	0·79 (0·69–0·86)	0·79 (0·74–0·82)	1·6 (1·5–1·7)	4·7 (3·2–7·3)
RISK6	0·77 (0·72–0·82)	0·86 (0·81–0·90)	0·45 (0·38–0·53)	0·65 (0·60–0·71)	0·73 (0·64–0·81)	0·72 (0·67–0·76)	1·5 (1·4–1·7)	3·7 (2·8–5·2)
Suliman4	0·73 (0·68–0·78)	0·78 (0·72–0·83)	0·54 (0·47–0·61)	0·67 (0·61–0·73)	0·67 (0·59–0·74)	0·63 (0·58–0·68)	1·5 (1·4–1·6)	3 (2·4–3·9)
C-reactive protein	0·61 (0·56–0·67)	0·83 (0·77–0·87)	0·35 (0·29–0·43)	0·61 (0·55–0·66)	0·63 (0·53–0·72)	0·75 (0·70–0·79)	1·7 (1·5–1·8)	2·7 (2·1–3·6)

Data are point estimates of each measure with 95% CI. Performance was indexed against the reference standard of positive culture or molecular test (Xpert MTB/RIF or Xpert MTB/RIF Ultra, excluding trace results) on samples from the site of disease or sputum. Sensitivity, specificity, PPV, NPV, triage positive rate, and NNT for each positive or negative case were calculated at the threshold of two SDs above the mean of the healthy latent tuberculosis control population for the blood RNA biomarkers and 10 mg/L threshold for C-reactive protein. p<0·0001 for all pairwise AUROC comparisons to C-reactive protein. AUROC=area under the receiver operating characteristic curve. NNT=number needed to test. NPV=negative predictive value. PPV=positive predictive value.

## Research in context

### Evidence before this study

Blood RNA biomarker signatures and C-reactive protein measurements have emerged as potential triage tests for tuberculosis, but evidence is mostly limited to their performance in pulmonary tuberculosis. Microbiological diagnosis of extrapulmonary tuberculosis is made challenging by the need for invasive sampling from the site of disease. This is compounded by lower sensitivity of confirmatory molecular tests for extrapulmonary tuberculosis compared with their performance in pulmonary disease. We did a systematic review of diagnostic accuracy studies of blood RNA biomarkers or C-reactive protein measurements for extrapulmonary tuberculosis, which could mitigate the need for site-of-disease sampling for the diagnosis of tuberculosis. We searched PubMed limited to English language publications from database inception to Jan 16, 2025, using the following search terms: “extrapulmonary [title/abstract] AND tuberculosis [title/abstract] AND biomarker [title/ abstract]”. Although extrapulmonary tuberculosis was included in several studies, none focused specifically on extrapulmonary tuberculosis or included an adequate number of individuals with extrapulmonary tuberculosis to provide precise estimates of test accuracy.

### Added value of this study

To our knowledge, we report the first diagnostic accuracy study of blood RNA biomarkers and C-reactive protein for tuberculosis among people with extrapulmonary tuberculosis. We examined the performance of seven previously identified blood RNA biomarkers as triage tests for tuberculosis lymphadenitis and tuberculosis pericarditis compared with a microbiology reference standard among people referred to hospital for invasive sampling in a setting with high tuberculosis and HIV prevalence. Multiple blood RNA biomarkers showed similar diagnostic accuracy to that previously reported for pulmonary tuberculosis in both extrapulmonary tuberculosis disease cohorts, irrespective of HIV status. All seven blood RNA biomarkers showed superior diagnostic accuracy to C-reactive protein for both lymphadenitis and pericarditis, but did not meet the combined characteristics of 90% or more sensitivity and 70% or more specificity recommended for a blood-based diagnostic triage test by WHO. Nonetheless, in decision curve analysis, using the best-performing blood RNA biomarker to trigger confirmatory microbiological testing showed superior clinical utility if the health-care service is seeking to reduce the number of invasive tests to fewer than four for each extrapulmonary tuberculosis case detected. If acceptable to undertake invasive testing in more than four people for each true case detected, then a test-all approach will provide greater net benefit in this tuberculosis and HIV hyperendemic setting.

### Implications of all the available evidence

Blood RNA biomarkers show potential as diagnostic triage tests for tuberculosis lymphadenitis and tuberculosis pericarditis, but do not provide the level of accuracy for blood-based triage tests recommended by WHO for community-based tests. C-reactive protein has inferior diagnostic accuracy to blood RNA biomarkers and cannot be recommended for diagnostic triage among people with extrapulmonary tuberculosis syndromes referred for invasive sampling.

## Data sharing

Processed Nanostring and C-reactive protein data with accompanying metadata are provided in appendix 2.
